# Perinatal γδ T cells: between invariance and diversity

**DOI:** 10.3389/fimmu.2026.1869398

**Published:** 2026-07-10

**Authors:** Rixa-Mareike Köhn, Immo Prinz

**Affiliations:** Institute of Systems Immunology, Hamburg Center for Translational Immunology, University Medical Center Hamburg-Eppendorf, Hamburg, Germany

**Keywords:** antiviral immune responses, congenital infection, neonates, perinatal γδ T cells, public TCR, γδ T cells, γδ TCR repertoire

## Abstract

The perinatal period represents a unique immunological window of opportunity, in which γδ T cells play a central role. New methods, including single-cell and high-resolution TCR repertoire analyses, have transformed our understanding of how γδ T cells develop, diversify, and function from fetal life into adulthood. Early γδ T cell waves display TCRs shaped by low TdT activity, biased V(D)J recombination, and favored V-J and V-D pairings that give rise to public, near germline-encoded TCRs with preprogrammed effector functions. These fetal-derived populations contribute to rapid pathogen responses and unique pathogen-driven expansions during congenital infections, revealing fundamental differences between perinatal and adult γδ T cell immunity. However, it is less clear how pre- and perinatal γδ T cells persist and influence individual immune responses later in life. This review synthesizes current insights into ontogeny, effector programming, and repertoire dynamics of γδ T cells in early life. We focus on deciphering the evident changes in the γδ T cell compartment during gestation, birth, and early life in humans.

## 1 Introduction

Early life represents a distinct immunological window that differs substantially from adult immune responses. The fetal and neonatal immune system depends strongly on the innate immune system, since adaptive immunity is not fully developed and not yet primed for extra-uterine life (1–3). This limited adaptive capacity is thought to contribute to the heightened susceptibility of fetuses and newborns to infections such as cytomegalovirus (CMV) (4, 5), toxoplasmoses (6), or malaria (7). During this developmental window, γδ T cells take on a pivotal role: they are the first T cells to develop and function at the interface of innate and adaptive immunity, providing rapid, pre-programmed effector responses (8, 9).

The early appearance of γδ T cells in ontogeny has been conserved in essentially all jawed vertebrate species, except squamata, for more than 450 million years (10, 11). The γδ T cell lineage is absent in squamates, suggesting that evolutionary selection may have promoted functional compensation between different lymphocyte lineages in some species (12). Akin to B and αβ T lymphocytes, γδ T cells generate their T cell receptor (TCR) through the rearrangement of variable (V), diversity (D; only *TRD*), and joining (J) gene segments. Theoretically, the possible rearrangements and combinations of *TRG* (γ-chain) and *TRD* (δ-chain) loci even exceed the diversity of αβ TCRs (13, 14). The number of possible TRD gene rearrangements is estimated at about 10^13^ because several D segments can be used during VDJ recombination. When this is combined with the additional diversity from more than 10^4^ TRG gene pairings, the total possible diversity of γδ TCRs is estimated at around 10^17^ (15, 16). In reality, however, γδ T cells often do not make use of their full recombination potential and frequently express (semi-)invariant TCRs. An invariant TCR is one whose TCR sequence is encoded by near-germline nucleotide sequences after V(D)J rearrangement, with no or minimal alterations. A semi-invariant TCR usually refers to a TCR with one invariant chain (e.g., the γ-chain) that pairs with a more diverse chain (e.g., the δ-chain). If TCR sequences are shared between individuals, the TCR is referred to as “public”, which occurs more frequently for invariant TCR sequences, in contrast to “private” sequences, which are not shared.

Unlike conventional αβ T cells, γδ T cells do not require MHC-presented antigens for ligand recognition via their TCR. To date, however, our knowledge about γδ TCR-ligand recognition remains fragmented (17, 18). γδ T cells can recognize a wide range of ligands, including classical and non-classical MHC molecules (19, 20), but γδ TCRs also engage in antibody-like recognition of chemically diverse molecules. A semi-invariant γδ TCR, using a combination of the Vγ9 chain and JP joining segment rearrangement and more diverse Vδ2 chains, reacts to non-peptide antigens, called phosphoantigens (pAgs) (21, 22). This recognition depends on butyrophilin family members, which are membrane proteins belonging to the immunoglobulin superfamily. In particular, BTN3A1 and BTN2A1 activate Vγ9Vδ2^+^ cells by mediating intracellular pAg sensing (23–25). In humans, Vγ9Vδ2^+^ T cells constitute the major γδ T cell population in adult peripheral blood and exhibit rapid, innate-like activation (26). In contrast, γδ TCR repertoire analyses show that many individuals possess large, persistent clonal expansions among non-Vγ9Vδ2 γδ T cells (27, 28), consistent with a more adaptive-like mode of immune responsiveness in these subsets.

Recent technological advances, for example, single-cell RNA-sequencing (scRNA-seq) and multi-omic approaches (e.g., scRNA-seq coupled with scTCR-seq, spatial transcriptomics, epigenomic profiling), have fundamentally transformed the understanding of human γδ T cell development, heterogeneity, and TCR repertoire dynamics. Although the availability of fetal and neonatal samples is restricted, these approaches now allow detailed insight into how γδ T cells emerge, diversify, and adapt from fetal life into adulthood. While two recent reviews have focused on the functional spectrum of γδ T cells in health and disease during the perinatal period (29) and across the life span (30), we focus on γδ T cell ontogeny and TCR repertoire formation during the perinatal period and their subsequent maturation. We highlight how neonates generate diverse γδ TCR repertoires and adaptive-like TCR specificities, despite the higher prevalence of near germline-encoded and (semi-)invariant γδ TCRs.

## 2 Development of γδ T cells in the perinatal thymus

### 2.1 Developmental waves of γδ T cells

The development of γδ T cells in thymic waves is well established for mice (31, 32). In short, several waves of γδ T cell progenitors occur, beginning early in embryogenesis, with characteristic TCR usage that determines their tissue homing. The first example is Vγ5Vδ1^+^ dendritic epidermal γδ T cells (DETCs), which are exclusively found in mice and home to the skin (33). Their progenitors are found solely in fetal and perinatal thymi, but DETC cells persist after birth as long-lived effector cells in the tissue (8). During thymic development, DETC cells are programmed to become IFN-γ-producing cells. This is driven by strong signaling through their public, invariant Vγ5Vδ1^+^ TCR in response to the selecting ligand Skint1, which is expressed on thymic epithelial cells (8, 34). Numerous aspects of γδ T cell thymic development differ substantially between mice and humans. Some of these differences include TCR rearrangement patterns, Notch signaling requirements, or fate decision windows (35). For example, developmental waves in mice are constrained by their *Trgv* usage, but less by their *Trdv* usage (36). In contrast, human γδ T cell waves are typically characterized by *TRDV* usage. It should be noted that there is a lack of definitive evidence concerning whether human γδ thymocytes rely on antigen-driven cues or ligand-independent TCR signaling, such as dimerization, during thymic selection and lineage commitment. In mice, TCR signal strength within specific developmental windows has been reported to be a key determinant of generating pro-inflammatory γδ T cell subsets, but whether a similar mechanism applies to humans remains unclear (35, 37).

Despite these similarities, important species-specific differences remain. In mice, several γδ T cell subsets acquire effector fate already during fetal thymic development, and some are maintained in tissues as long-lived, self-renewing populations (38). By contrast, the developmental origin of human γδ T cells is still less well defined. Although early fetal and neonatal human thymic and peripheral γδ T cells display clear signs of developmental programming, current evidence for a hematopoietic stem cell-independent origin of human γδ T cells remains limited. Recent studies of early human yolk sac hematopoiesis have identified lymphoid-biased progenitors before hematopoietic stem cell emergence, but these cells appear to contribute mainly to innate lymphoid and dendritic lineages rather than providing direct evidence for committed γδ T cell generation (39). At the same time, murine data show that at least some tissue-resident γδ T cell populations, such as dendritic epidermal T cells, can arise independently of definitive hematopoietic stem cells from yolk sac-derived hematopoiesis (40). Together, these findings suggest that while HSC-independent origins are established for certain murine γδ T cell populations, comparable evidence in humans is still lacking. Instead, human γδ T cells are thought to arise from thymus-seeding progenitors derived from fetal liver and later hematopoietic waves (35). These observations underscore that murine studies have provided important mechanistic insights into developmental timing, tissue imprinting, and effector programming, but direct extrapolation to human γδ T cell development remains limited by species-specific differences.

**Figure 1:**
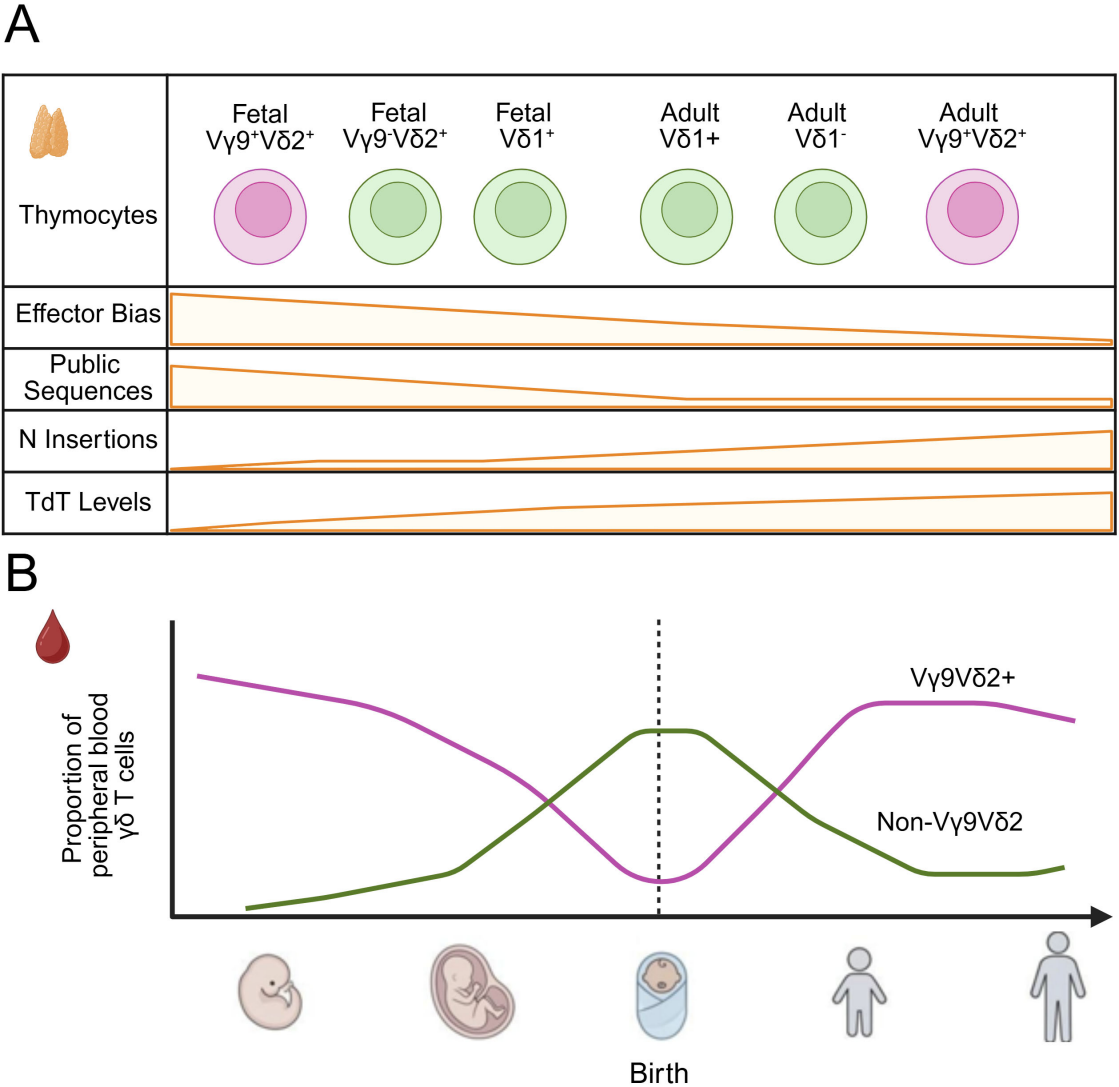
Development of human γδ T cells. The human γδ T cell compartment develops in distinct waves during fetal and postnatal life. **(A)** Fetal and postnatal γδ T cells differ in TCR features and effector programming. Early in gestation, low terminal deoxynucleotidyl transferase (TdT) activity reduces N insertions and junctional diversity, resulting in an enrichment of public sequences. Fetal thymocytes show a gestational age-dependent effector bias, whereas postnatal thymocytes are mainly naïve and enriched for private CDR3 sequences. **(B)** The proportions of Vγ9Vδ2^+^ and non-Vγ9Vδ2 cells in the peripheral blood vary across developmental stages. Non-Vγ9Vδ2 cells can predominate in the blood at birth. Vγ9Vδ2^+^ cells expand shortly after birth, whereas non-Vγ9Vδ2 cells often migrate into different tissues. Created in BioRender. Köhn, R. (2026) https://BioRender.com/b05duwu.

A wave-like development has also been discussed for humans, which is age-dependent and gives rise to γδ T cell subsets with a commitment to 3 different effector fates in the prenatal thymus, highlighting an effector bias early in gestation (10, 41) (**Figure 1A**). A first wave arises even before thymic development. Vγ9Vδ2^+^ cells appear as early as gestational week 5–6 in the fetal liver (42). Around gestational week 8-10, γδ T cells represent more than half of the developing T cells in the fetal thymus, out of which Vγ9Vδ2^+^ cells are the dominant subtype at this stage (43, 44). During the 2^nd^ trimester, Vγ9Vδ2^+^ cells are also the dominant subset in the blood (45) (**Figure 1B**). Subsequent to this initial wave, a population of Vδ2^+^ cells emerges in the thymus that pairs with other Vγ chains than Vγ9. These are discussed as a second Vδ2^+^ T cell wave and are most abundant in the thymus around mid-gestation (35, 46, 47). Because these cells display a broader *TRG* chain repertoire and lack responsiveness to pAgs, they are thought to function more similarly to Vδ1^+^ cells and exhibit more adaptive-like characteristics (48). For example, public fetal-derived Vγ9^-^Vδ2^+^ cells can, like fetal Vδ1^+^ T cells, expand upon and react to congenital CMV infection (5, 46).

Lastly, there is a prenatal wave of Vδ2^-^ γδ T cells, which includes Vδ1^+^ as well as less-studied Vδ3^+^ and Vδ5^+^ cells that pair with diverse γ-chains. Around gestational week 15 and 16, Vδ1 rearrangements dominate in the thymus (49). Vδ1^+^ T cells appear in the fetal blood around 25 weeks of gestation and are the major γδ T cell population at birth, together with an increase of Vδ3^+^ cells (5, 46, 50) (**Figure 1B**). After their initial enrichment in the blood around birth, non-Vγ9Vδ2 cells are thought to migrate predominantly into tissues, particularly mucosal sites (51, 52). Previous studies on human samples suggest that γδ T cells undergo tissue-specific adaptations shaped by the local environment, may contribute to tissue repair, and begin to acquire these adaptations early in life (51, 53). In the postnatal thymus, Vδ1^+^ cells are the predominant γδ T cell population (54, 55).

Closer to birth, increasing evidence suggests that adult waves of human Vδ1^+^ and Vδ2^+^ cells arise (10). A recent study reported that the postnatal thymus contains a small proportion of Vγ9Vδ2^+^ cell precursors that develop in three stages, defined by CD4 and CD161 expression, and exit the thymus as functionally mature cells in stage 3 (56). The authors propose that during infancy, the pool of postnatally developed Vγ9Vδ2^+^ expands through continued thymic output and peripheral proliferation. In line with this hypothesis, children who underwent neonatal thymectomy show an enrichment of fetal-derived Vγ9Vδ2^+^ T cells with cytotoxic characteristics, along with reduced TCR diversity of the Vδ1^+^ subset five to ten years after surgery (57). This notion of adult waves is further supported by the distinct effector programming of fetal and pediatric γδ thymocytes (9) (see section “Phenotypes and effector functions of early life and adult γδ T cells”), intrinsic biases in δ chain usage of CD34^+^ progenitor cells during development (46, 52), and age-dependent TCR rearrangement features (28, 47).

### 2.2 γδ TCR characteristics in early life

Fetal-derived γδ T cells can later be identified based on distinctive TCR characteristics. A main feature is a low number of N insertions, i.e. the lack of additional nucleotides in junctions during V(D)J gene segment joining, creating junctional diversity (14, 58). This process is controlled by the induction of terminal deoxynucleotide transferase (TdT, encoded by *DNTT*) in fetal thymocytes, which defines the lymphoid developmental trajectory from hematopoietic progenitors (46, 59). Tieppo et al. identified Lin28b, an RNA-binding protein, as a key player in controlling TdT expression, since overexpression in human stem cell progenitors (HSPCs) significantly downregulated *DNTT* expression and increased the number of invariant γδ TCR sequences (46). This is in line with a more general role of Lin28b in controlling fetal-like lymphopoiesis towards restricted generation of innate-like lymphocytes (60).

Low expression of TdT causes a reduction of N additions, which leads to the generation of invariant CDR3 sequences that are more likely to be shared between individuals. This could be confirmed in *in vitro* co-culture experiments as well as *in vivo* KO of *DNTT* in CD34^+^ progenitors in a humanized immune system mouse model (46, 52). The absence of TdT also promotes recombination events that rely on short homology repeats (61). It has been suggested that short, repeated nucleotide motifs within the germline-encoded V-J or V-D regions help explain why certain pairings are favored, giving rise to invariant CDR3 sequences. An example is the *TRGV8-TRGJP1* rearrangement, which produces the conserved sequence “CATWDTTGWFKIF,” observed in prenatal and neonatal samples (5, 46, 62, 63). At the same time, these short homology repeats can also contribute to combinatorial diversity, since even a single-nucleotide difference may shift the open reading frame. For example, there are two examples of *TRD* rearrangements leading to invariant sequences based on short homology repeats. This includes a *TRDV1* and *TRDV2* sequence rearranging with *TRDD3*, however, one leads to *TRDV1-TRDD3* “CALGELGD”, whereas *TRDV2-TRDD3* translates to “CACDTGGY” caused by a frame-shift (46). Notably, this reduced diversity appears independent of TCR-driven selection processes (61, 64), but they are conserved in both humans and mice (46). However, the specific sequence features that drive rearrangements remain only partly understood (61).

Preferential rearrangement patterns of J-segments can change with age. A preferred rearrangement with *TRGJP1* was identified in fetal thymocytes compared to *TRGJ1*, which was the most abundant J segment in the postnatal thymus (42). A systematic pattern analysis of Vδ2^+^ TCRs supported biased rearrangements of *TRDV2* with *TRDJ3* or *TRDJ2* in early life, which gradually shifted towards *TRDJ1* postnatally (65). Additionally, a “synonymous codon bias” was found, which describes multiple nucleotide transcripts encoding the same amino acid sequence. Similarly, Papadopoulou et al. (47) proposed that the germline-encoded *TRGV9-TRJP* CDR3 sequence “CALWEVQELGKKIKVF” can be encoded by alternative nucleotide sequences after V-J rearrangement, including N additions, in the postnatal thymus. Other factors that contribute to CDR3 diversity during V(D)J recombination include the insertion of P nucleotides, which are short palindromic sequences introduced when the hairpin coding ends are opened asymmetrically, and the trimming of nucleotides by exonucleases at the junction between two coding ends (47, 58). Possibly, age-dependent factors of the thymic microenvironment may also contribute to the generation of fetal-specific γδ T cells, as recently reviewed (10). Together, these developmental processes shape the γδ T cell populations that emerge from the thymus and help define their phenotypic and functional diversity. The γδ T cell subsets arising from each wave are summarized in **Figure 1A**.

## 3 Phenotypes and effector functions of early life and adult γδ T cells

Mirroring the unique developmental contexts of the fetal and pediatric thymus, γδ thymocytes from these stages display strikingly different and diverse effector programming. A landmark study highlighted the ability of single-cell RNA sequencing to resolve shared and subset-specific cytotoxic hallmarks of human Vδ1^+^ and Vδ2^+^ γδ T lymphocytes or tissue-specific adaptations of murine Vγ6^+^ γδ T cells, thereby providing a more nuanced and differential understanding of γδ T cell heterogeneity than, for example, flow cytometry-based analyses alone (66, 68). Due to these technical advances, approaches combining scRNA-seq with scTCR-seq now allow for a more detailed overview of particularly early life γδ T cell phenotypes (9, 69). Immature γδ T cells in the thymus are generally marked by CD1a expression (70). More mature cells in the fetal thymus showed functional programming that mirrored either type 1 (cytotoxicity, e.g., *Tbet*, NKG receptor, *IFN-γ*), type 2-like (e.g., *PLZF, CCR4, IL-2*), or type 3 immunity (e.g., *RORγt, CCR6, CD161, IL-17*) (9). Both Vγ9Vδ2^+^ and non-Vγ9Vδ2 T cells were represented in all three effector fates; however, an enrichment of public Vδ2 sequences was identified in mature effector clusters. Generation of effector cells was higher earlier in gestation, together with type 3 programming (9). Interestingly, this type 3 fetal subset may persist into adulthood, as a matching cluster with fetal-like TCR features could be identified in neonatal and adult blood samples, but no IL-17 transcripts were observed in these cells (69).

Thymocytes from pediatric origin comprised mostly naïve cells, but also smaller subsets of mature Vγ9Vδ2^+^ cells with type 1 and 3 effector signatures, and Vδ1^+^ cells expressing natural cytotoxicity receptor Nkp30 were present (9). Potentially, the latter give rise to adult anti-cancer Vδ1^+^ cells that further mature in the periphery after leaving the thymus (9, 55, 71). When γδ T cells, isolated from neonatal and adult blood samples, were analyzed, the two populations clustered distinctly by age (69). Cord blood-derived Vδ2^+^ cells, similar to fetal thymocytes, showed gene expression patterns associated with type 2-like immunity. Moreover, both type 1 and type 3 Vγ9Vδ2^+^ cells were detected in cord blood and adult blood, and an additional adult effector Vδ1^+^ subset was identified (69). Pediatric mature Vγ9Vδ2^+^ cells with dual effector functions likely represent stage 3 (CD4^-^ CD161^+^) cells that developed postnatally in the thymus (56).

Perinatal γδ T cells can elicit rapid immune responses against viral threats *in utero*. For example, congenital CMV infection causes the expansion of fetal-like non-Vγ9Vδ2 cells displaying a cytotoxic phenotype and showing reactivity against CMV-infected cells *in vitro* (5, 62, 63). Although not ascribed to a certain TCR-holding clone, a recent study reported an increase of IFN-γ- or TNF-α-producing Vδ2^+^ cells in the cord blood of neonates born to HIV^+^ women (72). Another study suggested more potent IFN-γ production after activation of neonatal γδ T cells compared to their αβ counterparts (73). This is in contrast to limited functionality compared to adult γδ T cells. For example, *ex vivo* experiments with influenza viruses suggested limited cytotoxic functions of neonatal γδ T cells compared to healthy adult Vγ9Vδ2^+^ cells (74). However, fetal Vγ9Vδ2^+^ cells hold potent effector capabilities even before encountering pathogens and expand polyclonally shortly after birth (45, 75). Furthermore, Vγ9Vδ2^+^ cells expand as potent cytotoxic effector cells in response to congenital (phosphoantigen-producing) *Toxoplasma gondii* infection (6, 76). Notably, placental malaria has an effect on both Vγ9Vδ2^+^ and non-Vγ9Vδ2 subsets (7, 77). Together, fetal and perinatal γδ T cells are important components of early-life immunity during infectious diseases beyond the examples mentioned. Their functional spectrum includes, besides cytotoxicity and cytokine production, immune regulation and tissue homeostasis (29).

While evidently, non-Vγ9Vδ2 γδ T cells are disseminated across tissues holding important site-specific functions (78–80), the functionality of perinatal γδ T cells in tissues is poorly understood. Availability of neonatal and pediatric tissue is highly limited, which is why our understanding of site-specific adaptation and tissue residency hallmarks of γδ T cells is mostly based on sophisticated mouse models (81). An extensive study of blood and tissues from 176 donors across the lifespan gave valuable insight into perinatal (and pediatric) γδ T cell functions in different tissues (51). Compared to adult tissues, γδ T cells are significantly enriched in spleen, lung, and jejunum. Overall, Vδ1^+^ cells rather displayed tissue-repair functions (AREG^+^), whereas Vδ2^+^ (mostly Vγ9^+^), showed more effector-like and cytolytic profiles, especially in the lung (51). However, the authors suggested that naïve Vδ1^+^ and Vδ2^+^ cells are established in tissues early in life and then progressively differentiate into effector and memory T cells in a site-specific manner throughout childhood. Beyond the role of perinatal γδ T cells in infection and tissue homeostasis, it is likely that these cells also contribute to early development of autoimmunity, for example, juvenile rheumatoid arthritis (82), or cancer, such as pediatric neuroblastoma (83). However, only very few studies have elucidated the contributions of perinatal (or pediatric) γδ T cells in these disease contexts so far.

Supposedly, the public near germline-encoded TCRs of perinatal γδ T cells may have developed or persisted because of co-evolution with pathogens that pose a threat *in utero* or early in life, or because of tissue-specific functions during development. Intriguingly, the impact of different diseases and environmental cues on the γδ TCR repertoire varies across the lifespan (see next section “Repertoire dynamics of perinatal γδ T cells across the lifespan”). However, variation in depth of subset definition (e.g., Vδ2^+/-^ vs. Vγ9Vδ2^+^ and non-Vγ9Vδ2) and technical limitations, such as restricted sample availability and cohort variability, limit our in-depth understanding of subset differences within the perinatal γδ T cell compartment. Additionally, there is a bias in current studies of perinatal γδ T cell functionality towards congenital CMV.

## 4 Repertoire dynamics of perinatal γδ T cells across the lifespan

### 4.1 Age-related repertoire changes

**Figure 2:**
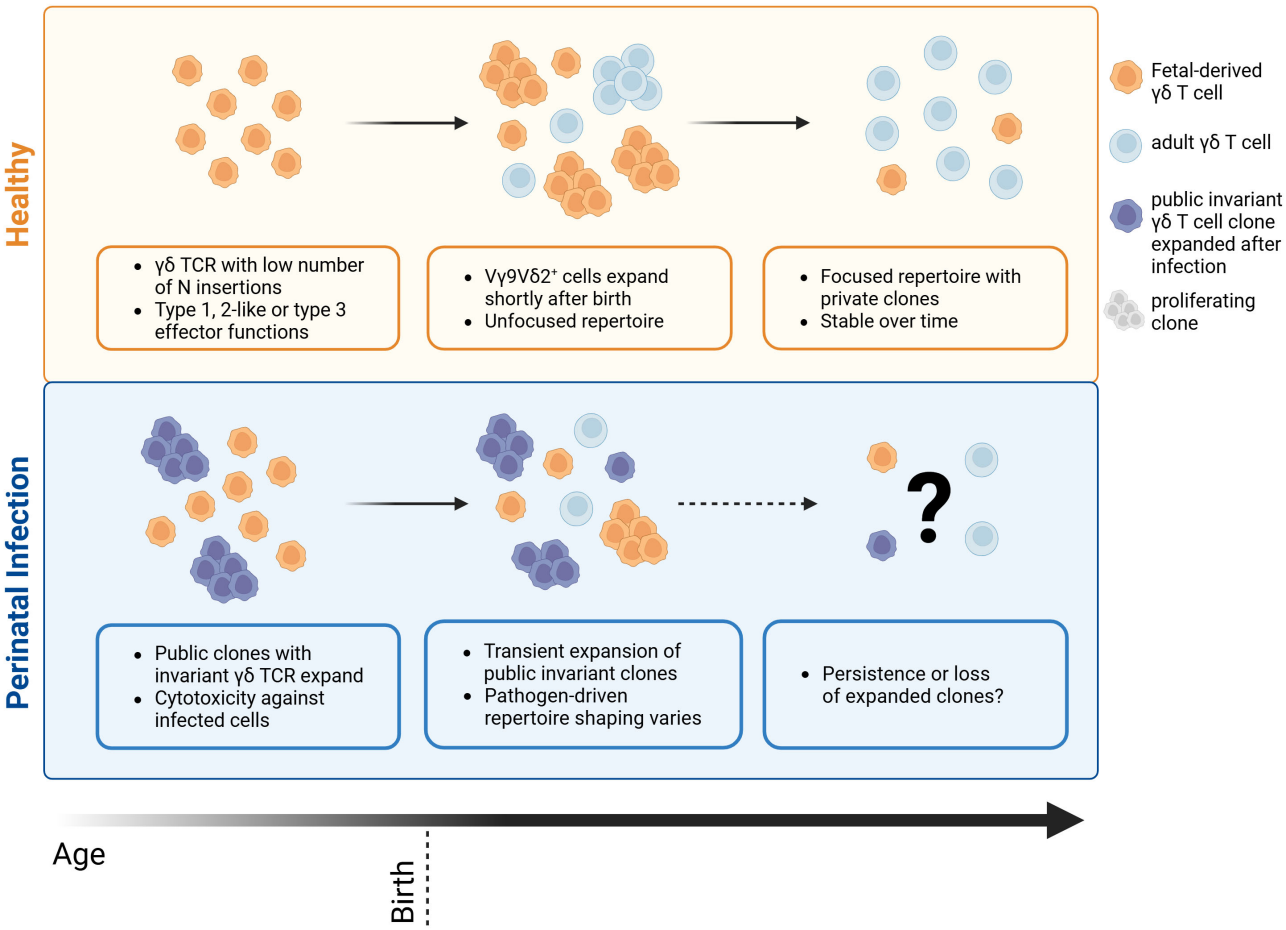
Perinatal γδ T cells across the lifespan. During fetal development, thymic γδ T cells preferentially express γδ TCRs with little or no N additions and more frequently public CDR3 sequences. Fetal γδ T cells exhibit a bias towards preprogrammed effector functions according to a type 1, type 2-like or type 3 immune response. Shortly after birth, Vγ9Vδ2^+^ cells carrying public Vγ9-JP rearrangements expand in the periphery. In addition, adult-like Vγ9Vδ2^+^ cells exit the postnatal thymus. The adult γδ TCR repertoire is focused and largely composed of private clones. In response to perinatal infection, public fetal γδ T cell clones expand that frequently display a cytotoxic phenotype. The specific γδ T cell subset that expands varies between pathogens. For example, CMV causes an expansion of non-Vγ9Vδ2 cells, whereas Toxoplasma gondii infection or neonatal sepsis causes an expansion of specific Vγ9Vδ2^+^ cells. These expansions appear transient and do not perturb γδ T cell profiles across infancy. Whether such clones persist into adulthood or disappear entirely from the adult γδ repertoire remains unclear. The image was created in BioRender. Köhn, R. (2026) https://BioRender.com/t5viiqm.

The γδ TCR repertoires of healthy neonates or pediatric samples are reported as unfocused or polyclonal, while adult repertoires are referred to as less diverse or more “focused” with private oligoclonal expansions and γδ TCRs encoded with a higher number of N additions (27, 28, 55, 84, 85) (**Figure 2**). Interestingly, an increase in N additions of the Vδ2 repertoire was already noticeable in fetal thymocytes from 21 weeks of gestation onwards (9). Especially paired Vγ9Vδ2^+^ cells may undergo postnatal expansion and phenotypic maturation of fewer fetal-derived clones that persist into adulthood (**Figure 2**), and function besides adult Vγ9Vδ2^+^ cells that developed closer to birth or postnatally (47, 69, 75, 86–89). It is not fully elucidated whether the expansion of Vγ9Vδ2^+^ cells is dependent on antigen exposure; however, polyclonal expansion of these cells after birth may be partly driven by microbial encounter (75, 87, 90). Since mode of delivery and antibiotic treatment did not affect Vγ9Vδ2^+^ expansion after birth, other factors likely contribute to this expansion (88). In addition, postnatal thymic output of Vγ9Vδ2^+^ cells may partially lead to an increase in numbers (56). Further studies are needed to determine more definitively whether peripheral expansion is driven predominantly by fetal-derived or adult Vγ9Vδ2 T cells, under which conditions this expansion occurs, and how relevant postnatal thymic output of these cells truly is. Allegedly, later in life, a decline of Vγ9Vδ2^+^ cells in absolute numbers and frequencies could promote a proinflammatory state in the elderly (91). This is concomitant with a shrinking of the naïve γδ T cell population and a decrease in Vγ9 usage of effector and central memory γδ T cells upon ageing (92, 93).

Due to the presence of the public Vγ9-JP chain, the adult *TRG* repertoire is more public than the *TRD* repertoire (27, 94). However, when *TRG* repertoires from adults and cord blood samples were compared, cord blood showed an even higher proportion of shared clones between individuals (27), highlighting the prevalence of public sequences early in life. In contrast, the *TRD* repertoire in healthy adults was almost entirely private. The Vδ1^+^ TCR repertoire is focused on a few dominant and private clones, while this oligoclonal expansion was not observed in neonates (28). These expanded clones were described as long-lived and differentiated (decrease in CD27 expression). Generally, the γδ TCR repertoire evaluated in the blood remains stable over time; specifically, Vδ1^+^ populations remain stable even in elderly samples (27, 28, 95). The γδ TCR repertoire also reconstitutes rapidly after stem cell transplantation, but its composition is described as fundamentally different from that observed prior to transplantation (27). Due to the very limited availability of human tissue samples, especially in infancy, data on γδ TCR repertoires in tissues remain sparse. Generally, Vδ1^+^ γδ T cells dominate in all organs in infancy except the lung (53). In pediatric samples, both *TRD* and *TRG* repertoires were diverse across blood, spleen, and lymph nodes and were more expanded at mucosal sites (51). The study reported minimal clonal overlap between *TRD* clones across organs, which increased over childhood to levels observed in adult samples. In conclusion, a more “focused” Vδ1 and Vδ2 repertoire likely disseminates across tissues with age-dependent clonal expansions.

While repertoire analyses of γδ T cells are clearly relevant for further elucidating the biology and functions of this enigmatic cell type, such approaches require a rigorous definition of γδ T cells that is not based solely on transcriptomic signatures. This is particularly important in large scRNA-seq datasets, where technical limitations, including dropout of TCR transcripts and incomplete capture of variable TCR regions, can hinder accurate identification (96). In addition, TRG transcripts may overlap with αβ T cells, further complicating transcript-based classification. Moreover, populations of γδ T cells expressing an extensive repertoire of NK cell receptors, as well as conventional NK cells displaying germline TCRδ transcripts, further highlight the close relationship between NK cells and T cells and the challenges of discriminating between these lineages (97). Accordingly, reliable γδ T cell annotation ideally requires complementary information from TCR sequencing or protein-based receptor detection.

### 4.2 Pathogen-driven repertoire changes

Antigen challenge is thought to induce focusing of the γδ TCR repertoire throughout life. Healthy elderly individuals show clonal expansion of non-Vγ9Vδ2 cells, which is thought to reflect past antigen challenges (74), but not all healthy adults show expanded Vδ1^+^ clones (28). It is still unclear which specific antigenic challenges, and under what conditions, lead to definitive changes in the clonotypic composition of γδ T cells. Pathogen-driven influences on the γδ TCR repertoire can be very nuanced, as some, but not all, pathogens that lead to sepsis strongly influenced the Vδ2 repertoire, and only in patients aged two years or younger (98). Similarly, clearing a persistent hepatitis C infection in adults did not alter the γδ T cell repertoire (99). Detailed analyses of repertoire dynamics following strong antigen challenges remain limited, especially during the neonatal period. One study established a longitudinal cohort of preterm neonates with neonatal sepsis and healthy controls at four timepoints, comparing them to healthy term infants (88). They found an enrichment of fetal-derived Vγ9Vδ2^+^ cells in neonates with sepsis, while fetal-like *TRDV* chain abundance was similar between preterm and term infants at 13–16 and 12–24 months, respectively. *TRD* repertoire diversity showed no significant differences related to sepsis at any timepoint. Important pathogenic challenges during pregnancy and early life include, besides sepsis, HIV (72, 100), malaria (7, 77), *Toxoplasma gondii* (6) or CMV (5, 62, 63). The impact of early vaccination also remains insufficiently understood. Notably, a measles vaccine showed no effect on the *TRD* repertoire (75).

CMV infection or reactivation may be the most thoroughly characterized infection affecting the γδ TCR repertoire and eliciting γδ T cell functions to date (101). Adoptive γδ T cell therapy even controls CMV infection in preclinical models, but independent of γδ TCR recognition (102). In adults, CMV infection and reactivation (27) generally lead to an expansion of mostly Vδ1^+^ cells, which are private and not shared between individuals, but persist for months (27, 28). However, clonal expansion of Vδ1^+^ γδ T cells has also been observed in CMV-seronegative adults, suggesting that other immune challenges could be responsible for this expansion (28). Vδ1^+^ cells expanded after CMV infection are not necessarily reactive to CMV-encoded ligands, but rather, they are more likely to react to CMV-induced ligands. For example, one expanded Vδ1^+^ clone that derived from a patient with CMV reactivation after stem cell transplantation recognized HLA-DR (19). This is in line with the idea that γδ T cells recognize cellular dysregulation and stress signals, rather than pathogen-specific antigens, explaining an overlap of anti-viral and anti-cancer activities of γδ T cells (103). As described above, *in utero* CMV infection caused an expansion of a shared invariant Vγ8Vδ1^+^ clone in infected individuals. This clone was also identified in other studies of congenital CMV infection; however, not in all CMV^+^ individuals, and only in infants <120 days of age (62, 63). More public near germline-encoded non-Vγ9Vδ2 clones that expanded after *in utero* CMV infection were described, but the Vγ8Vδ1^+^ clone had the highest level of publicity among CMV^+^ individuals (62). To our knowledge, these near germline-encoded clones are not reported in any adult studies of CMV infection. When the presence of the public Vγ8Vδ1^+^ clone was specifically traced by PCR, this clone was undetectable in CMV^+^ neonates after two months of infection (63). These data indicate a fundamental difference in the response of γδ T cells to CMV infection in perinatal and adult life. Seemingly, protection against perinatal infection strongly depends on limited, near germline-encoded diversity of γδ TCR clones that do not persist (in the blood) over a long period of time (**Figure 2**). Whether these clones persist in the γδ TCR repertoire, for example, in tissues, remains open to speculation. Besides a cohort of neonatal sepsis (88), no longitudinal study from birth to childhood (or adulthood) of the same individuals has been published. Tracking γδ TCR dynamics in individuals longitudinally, especially from birth through adulthood, could yield richer insight into the complex developmental trajectories of γδ T cells and their persistence.

## 5 Significance of limited junctional diversity and near germline-encoded γδ TCRs

If V(D)J recombination were truly random, or even semi-random, public TCR chain repertoires should be a rare event (104). This is especially true if paired TCR chains are shared between individuals or if no MHC interaction is required for antigen recognition. Nevertheless, semi-invariant Vγ9Vδ2^+^ TCRs are shared across individuals, and congenital CMV infection can lead to the expansion of highly public Vγ8Vδ1^+^ T cells. The paradox of public TCR responses has prompted several hypotheses, which were discussed in detail by Venturi et al. (105). These include (1) structural explanations, (2) sequence-based explanations, and (3) convergent recombination (see **Table 1** “Three different hypotheses explaining public (γδ) TCRs”).

**Table 1:** Three different hypotheses explaining public (γδ) TCRs.

Explanation for public (γδ) TCRs	Example V chains	Example CDR3 sequence	Explanation	Reference
Structural	Vγ9-JP	CALWE(V)QELGKKIKVF	Structural constraints causing coevolution of butyrophilin-binding pAG sensing Vγ9 chains	(24, 45, 47)
Sequence-based	Vγ8Vδ1	γ CATWDTTGWFKIF δ CALGELGDDKLIF	Increased likelihood for germline-encoded sequences containing short homology repeats in fetal thymi	(5, 46, 63)
Convergent Recombination	Vγ9Vδ2	γ CALWEVQELGKKIKF δ CACDTLGDTDKLIF	Synonymous codon bias encoding the same sequence as germline	(47, 65)

One proposed explanation is that public Vγ9-JP TCRs may arise due to structural constraints required to interact with butyrophilins, such as BTN2A1, which could confer a selection bias favoring chains capable of phosphoantigen sensing, as already suggested by Fichtner et al. (106). Convergent recombination might also explain the presence of Vγ9Vδ2^+^ T cells, encoded by both germline sequences and nucleotide sequences with N additions, which was originally termed a “synonymous codon bias” by Deng et al. (65). Supporting this idea, the enrichment of hydrophobic residues at position five of the *TRDV2* CDR3δ paired with Vγ9 chains correlates with phosphoantigen reactivity (52).

Near germline-encoded TCRs may be more common because of reduced N additions and limited V-J and V-D pairing, directed by short homology repeats (61). This decreases the diversity of possible recombinations, thus increasing the likelihood that certain sequences appear across individuals. Such a developmental bias could also account for the higher prevalence of public (non-Vγ9Vδ2) T cell clones in neonates compared to adults, a difference potentially driven by lower TdT activity early in life and intrinsic differences in stem cell progenitors.

Still, many questions remain: Why are specific γ and δ chain pairings, such as the Vγ8Vδ1^+^ clone, favored? What evolutionary advantages do these near germline-encoded receptors confer specifically during early life infections? Is there a selection mechanism that makes clones like Vγ8Vδ1^+^ even more likely to expand? Tuengel et al. (63) hypothesized that this Vγ8Vδ1^+^ clone exists in every individual at levels below detection, expanding notably in cases of congenital CMV infection. Interestingly, the public Vγ8^+^ chain alone also appears in the absence of CMV infection (46, 52, 94). The rapid disappearance of the Vγ8Vδ1^+^ clone from the blood following infection may be due to swift migration into tissues or perhaps because its cytotoxic reactivity poses (self-)harm if maintained. In case of tissue residency, it could be speculated that this clone might possess local memory- or stem-like functions akin to those described for γδ intraepithelial lymphocytes (80, 107). However, these functions would be limited to tissues, as this clone is not found in adult PBMCs after CMV infection or reactivation. The restricted selection of so far identified γδ T cell ligands suggests recognition of self-ligands rather than pathogen-induced ligands by the γδ TCR (17). How these γδ TCRs undergo thymic selection remains poorly understood. A study using transgenic mice suggested a window of weakened negative selection within the neonatal thymus, which led to an escape of auto-specific γδ T cells to the intestine (108). Whether fetal invariant γδ TCRs are indeed auto-reactive and whether an expansion of clones holding such γδ TCRs after perinatal infections has an impact on the occurrence of autoimmunity later in life can only be speculated and requires further research. Further, this raises the question of whether a reservoir of such near germline-encoded TCRs exists. It remains unclear whether highly public clonal populations could exist that behave similarly to the Vγ8Vδ1^+^ clone but respond to antigenic challenges other than CMV, potentially displaying polyspecificity as described for adult γδ T cells (109) and likewise suggested for CD8^+^ T cells (67). More research is needed to clarify the functions of these invariant TCR clones and their contribution to early-life immunity.

## 6 Conclusions

During the perinatal period, γδ T cells develop in the thymus with a bias toward “public” TCRs that are nearly germline-encoded, with favored V-J or V-D pairing, and preprogrammed effector functions, particularly before birth. These cells play an important role in mounting rapid responses to early-life challenges and may have co-evolved in response to threats such as CMV. The perinatal γδ T cell repertoire may function as a balance between evolutionary preparedness and early adaptive responses before adaptive immunity fully develops. Perinatal γδ T cell development reflects a trade-off between invariant, rapidly responding public TCRs and the gradual emergence of more private, refined repertoires. Germline-encoded γδ TCRs likely provide an initial layer of protection until individualized, more adapted γδ TCR repertoires mature later in life. Some fetal-like γδ T cell populations persist into adulthood, but it is mostly unclear how long they remain, where they reside, and how much they influence later immune responses. While effector functions of germline-encoded TCR clones were described, their defined physiological functions and TCR-specificity remain undetermined. How early-life immune challenges imprint the γδ TCR repertoire or influence the persistence of certain γδ T cell clones is still unknown. This underscores the need for longitudinal studies that track the γδ TCR repertoire within individuals over time. Further research is necessary to predict outcomes and successfully intervene in early-life immune challenges.
